# Deep learning-based optic disc classification is affected by optic-disc tilt

**DOI:** 10.1038/s41598-023-50256-4

**Published:** 2024-01-04

**Authors:** Youngwoo Nam, Joonhyoung Kim, Kyunga Kim, Kyung-Ah Park, Mira Kang, Baek Hwan Cho, Sei Yeul Oh, Changwon Kee, Jongchul Han, Ga-In Lee, Min Chae Kang, Dongyoung Lee, Yeeun Choi, Hee Jee Yun, Hansol Park, Jiho Kim, Soo Jin Cho, Dong Kyung Chang

**Affiliations:** 1 Medical AI Research Center, Institute of Smart Healthcare, Samsung Medical Center, Seoul, Republic of Korea; 2 Department of Digital Health, SAIHST, Sungkyunkwan University, Seoul, Republic of Korea; 3 Sungkyunkwan University School of Medicine, Seoul, Republic of Korea; 4 Biomedical Statistics Center, Research Institute for Future Medicine, Samsung Medical Center, Seoul, Republic of Korea; 5 Department of Data Convergence & Future Medicine, Sungkyunkwan University School of Medicine, Seoul, Republic of Korea; 6 Department of Ophthalmology, Samsung Medical Center, Sungkyunkwan University School of Medicine, 81 Irwon-ro, Gangnam-gu, Seoul, 06351 Republic of Korea; 7 Health Promotion Center, Samsung Medical Center, Sungkyunkwan University School of Medicine, 81 Irwon-ro, Gangnam-gu, Seoul, 06351 Republic of Korea; 8 Department of Medical Device Management and Research, SAIHST, Sungkyunkwan University, Seoul, Republic of Korea; 9 Digital Innovation Center, Samsung Medical Center, Sungkyunkwan University School of Medicine, Seoul, Republic of Korea; 10 Division of Gastroenterology, Department of Internal Medicine, Samsung Medical Center, Sungkyunkwan University School of Medicine, Seoul, Republic of Korea; 11 Department of Biomedical Informatics, CHA University School of Medicine, CHA University, Seongam, Republic of Korea

**Keywords:** Optic nerve diseases, Machine learning

## Abstract

We aimed to determine the effect of optic disc tilt on deep learning-based optic disc classification. A total of 2507 fundus photographs were acquired from 2236 eyes of 1809 subjects (mean age of 46 years; 53% men). Among all photographs, 1010 (40.3%) had tilted optic discs. Image annotation was performed to label pathologic changes of the optic disc (normal, glaucomatous optic disc changes, disc swelling, and disc pallor). Deep learning-based classification modeling was implemented to develop optic-disc appearance classification models with the photographs of all subjects and those with and without tilted optic discs. Regardless of deep learning algorithms, the classification models showed better overall performance when developed based on data from subjects with non-tilted discs (AUC, 0.988 ± 0.002, 0.991 ± 0.003, and 0.986 ± 0.003 for VGG16, VGG19, and DenseNet121, respectively) than when developed based on data with tilted discs (AUC, 0.924 ± 0.046, 0.928 ± 0.017, and 0.935 ± 0.008). In classification of each pathologic change, non-tilted disc models had better sensitivity and specificity than the tilted disc models. The optic disc appearance classification models developed based all-subject data demonstrated lower accuracy in patients with the appearance of tilted discs than in those with non-tilted discs. Our findings suggested the need to identify and adjust for the effect of optic disc tilt on the optic disc classification algorithm in future development.

## Introduction

Myopia and high myopia were estimated to affect 1893 million and 170 million people worldwide, respectively, in 2010^1^, and it is expected that the prevalence will significantly increase globally to nearly 5 billion people and 1 billion people, respectively, by 2050^2^. The prevalence is higher in Asia than in Western communities and is rising rapidly, especially in East Asia^2,3^. A tilted appearance of the optic disc, which has been reported to be closely related to myopia, is not infrequently found in clinical examinations^4,5^. A tilted optic disc can affect not only the appearance of the optic disc but importantly, the ocular parameters in optical coherence tomography and visual field analyzers, which, along with fundus photographs, are the most common tools used in ophthalmology clinics^6–11^. Therefore, with the rapid increase in the prevalence of myopia, ophthalmic diagnoses using imaging will become more challenging.

In recent decades, deep learning systems within artificial intelligence have been utilized for analyzing and diagnosing of various ophthalmologic diseases, including diabetic retinopathy, glaucomatous optic neuropathy, papilledema, and optic atrophy^12–19^. As it is a field that is highly dependent on imaging tests, ophthalmology has been in a prime position to witness the application of deep learning algorithms for analyzing the vast amount of data from those tests^12^. While deep learning usually requires a large amount of data for training, large image databases are not available in some ophthalmology fields such as neuro-ophthalmology^13^. However, despite the relatively limited data, previous studies revealed that deep learning algorithms performed as quickly and accurately or even better than expert ophthalmologists in the classification of optic nerve appearances including neuro-ophthalmic abnormalities^13–16^. Thus, these algorithms show promise for the automated interpretation of ophthalmologic images^16–19^.

Given the increasing prevalence of myopia, it is vital to determine how optic disc tilt influences the parameters of ophthalmologic tools, especially when establishing an automated ophthalmologic diagnostic or prognostic system. In this study, we developed deep learning-based optic disease classification systems using the ocular fundus photographs of patients with or without tilted optic discs and compared the classification performance between the systems. Our goal was to ascertain the extent and manner in which optic disc tilt impacts the accuracy of these systems.

## Methods

The Institutional Review Board of Samsung Medical Center (SMC, Seoul, Republic of Korea) approved this study and waived the requirement for informed consent given the retrospective nature of the study and all methods were performed in accordance with the relevant guidelines and regulations. All fundus photographs in the study were de-identified and the adequacy of data de-identification was assessed and approved by the Research Resources Standardization Center of SMC.

### Data collection and annotation

This study included healthy subjects and patients with optic neuropathies from Health Promotion Center and Department of Ophthalmology in SMC between January 2007 and December 2021. Retrospectively collected fundus photographs were acquired from one or both eyes of these subjects with the use of TRC-50IX digital camera (Topcon, Tokyo, Japan) or Kowa nonmyd 10-megapixel fundus camera (Kowa, Torrance, CA, USA) at various fields of view (20 to 50 degrees). We excluded photographs with low quality from the study, such as those with unclear optic disc or with excessive darkness.

Outcome class annotations with four labels (normal, glaucomatous optic disc change, disc swelling, and disc pallor) were made to provide deep-learning networks with reference standards for pathologic changes in optic discs. A fundus photograph was labeled as 'normal' when no ophthalmic diseases were previously diagnosed, barring mild media opacity. Additionally, the optic nerve had to be verified as normal by two neuro-ophthalmologists, corroborated by standard vision and intraocular pressure results.

Glaucomatous optic disc change was confirmed by two glaucoma specialists based on glaucomatous visual field defects on Humphrey 740 Visual Field Analyzer (Carl Zeiss Meditec Inc. Dublin, CA, USA), corresponding glaucomatous change in the optic disc, and peripapillary retinal nerve fiber layer (pRNFL) thinning in Cirrus high-definition optical coherence tomography (OCT) (Carl Zeiss Meditec AG, Jena, Germany), as well as follow-up evaluations.

Two types of optic-disc abnormalities were diagnosed by two neuro-ophthalmologists based on comprehensive reviews of the appearance of optic-nerve head as well as OCT, medical records, brain imaging, cerebrospinal fluid opening pressure, and follow-up visits: (a) optic disc swelling caused by intracranial hypertension, acute anterior ischemic optic neuropathy, or anterior inflammatory optic neuropathies, and (b) optic disc pallor caused by various optic neuropathies (compressive, ischemic, inflammatory, traumatic, toxic, or hereditary optic neuropathies) in the chronic stage. All optic disc swelling showed thickening with a mean pRNFL value above the normal limit, and all optic disc pallor showed thinning with mean or sectoral pRNFL values below the normal limit with *p* < 0.01 on Cirrus high-definition OCT or Spectralis spectral-domain OCT (Heidelberg Engineering, Heidelberg, Germany). Images from patients who both had glaucoma and other types of optic neuropathy were excluded.

Annotations on the tilt status (non-tilted and tiled) of optic discs were made by a neuro-ophthalmologist using ImageJ 1.48v (National Institute of Health, Bethesda, MD, USA). The ovality index was assessed as the ratio of maximal-to-minimal optic disc diameter. A significant disc tilt was identified with an ovality index of more than 1.3^20–22^. A typical non-tilted disc was defined as an optic disc in a normal optic nerve head shape without any semilunar patch of sclera around it. The annotations were cross-verified among three reviewers. Images with discrepancies were excluded from the study.

### Data preparation

**Figure 1:**
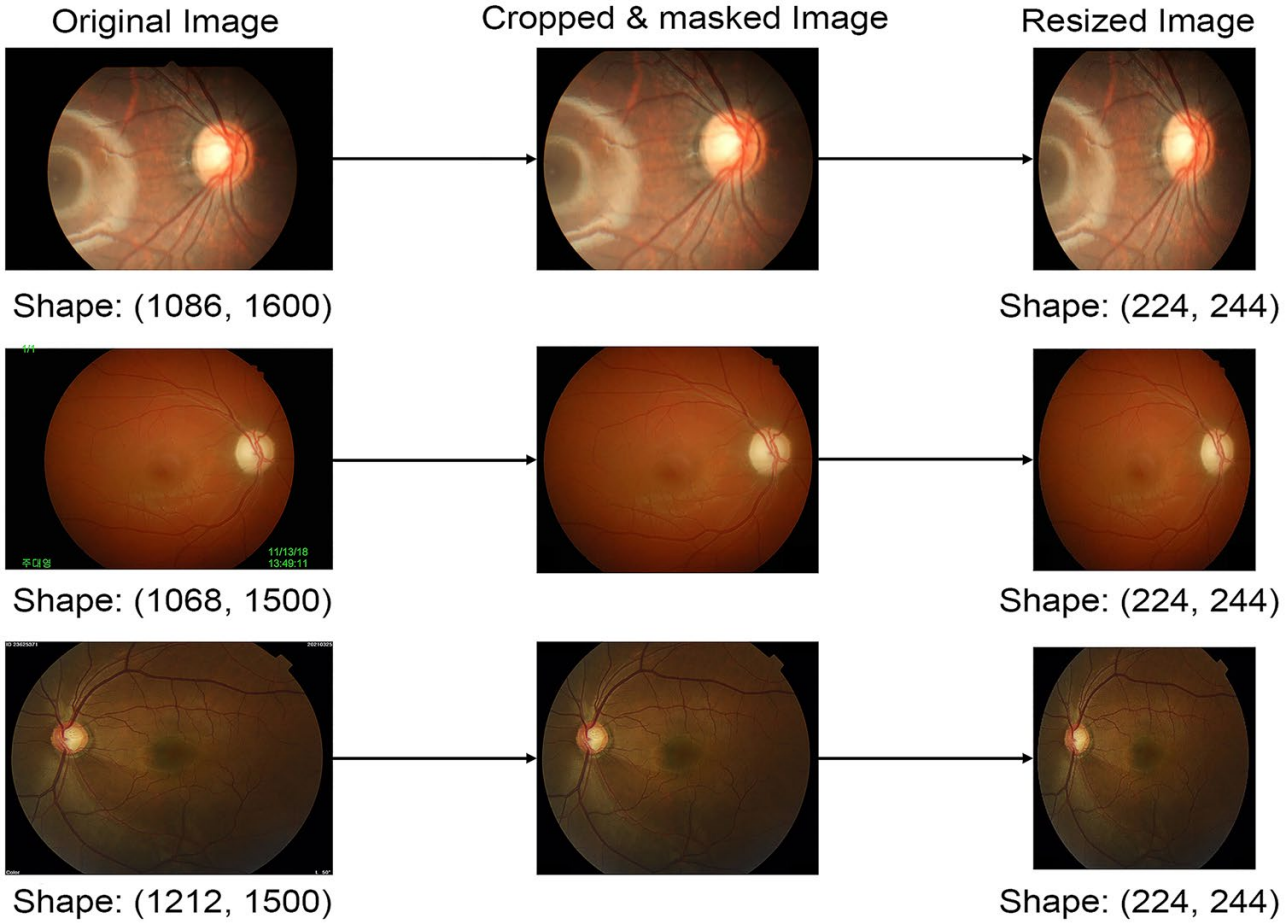
Masking and cropping process. The original images exhibited a range of sizes (left). Superfluous data, such as text, was masked out from the original images, and extraneous spaces were cropped (middle). The areas for masking and cropping in each image were manually determined by a human annotator. Following the preprocessing, images were resized to dimensions of (224, 224) (right).

The collected images with different sizes ranging from 709 × 748 pixels to 3000 × 3000 pixels, were processed prior to being trained using deep convolutional neural networks (CNNs) for optic disc classification (Fig. 1). We cropped images to eliminate unnecessary peripheral edges outside optic discs. All irrelevant information on the images were properly masked, including patient ID, date and shooting angles. All images were resized to the same size of 224 × 224 pixels, and pixel values were scaled from 0 to 1. When resizing an optic disc image, its original aspect ratio was not preserved because it would produce better performance^23^.

Data augmentation was applied to address the data insufficiency issue by adjusting the brightness, shifting the width, and flipping along the horizontal axis of the original images. The image pixel values were normalized by subtracting the mean and dividing by the standard deviation. This normalization ensured that each image had a consistent distribution, and thus promoted fast convergence during the deep-learning training procedure.

### Deep-learning algorithms for four-class classification

**Figure 2:**
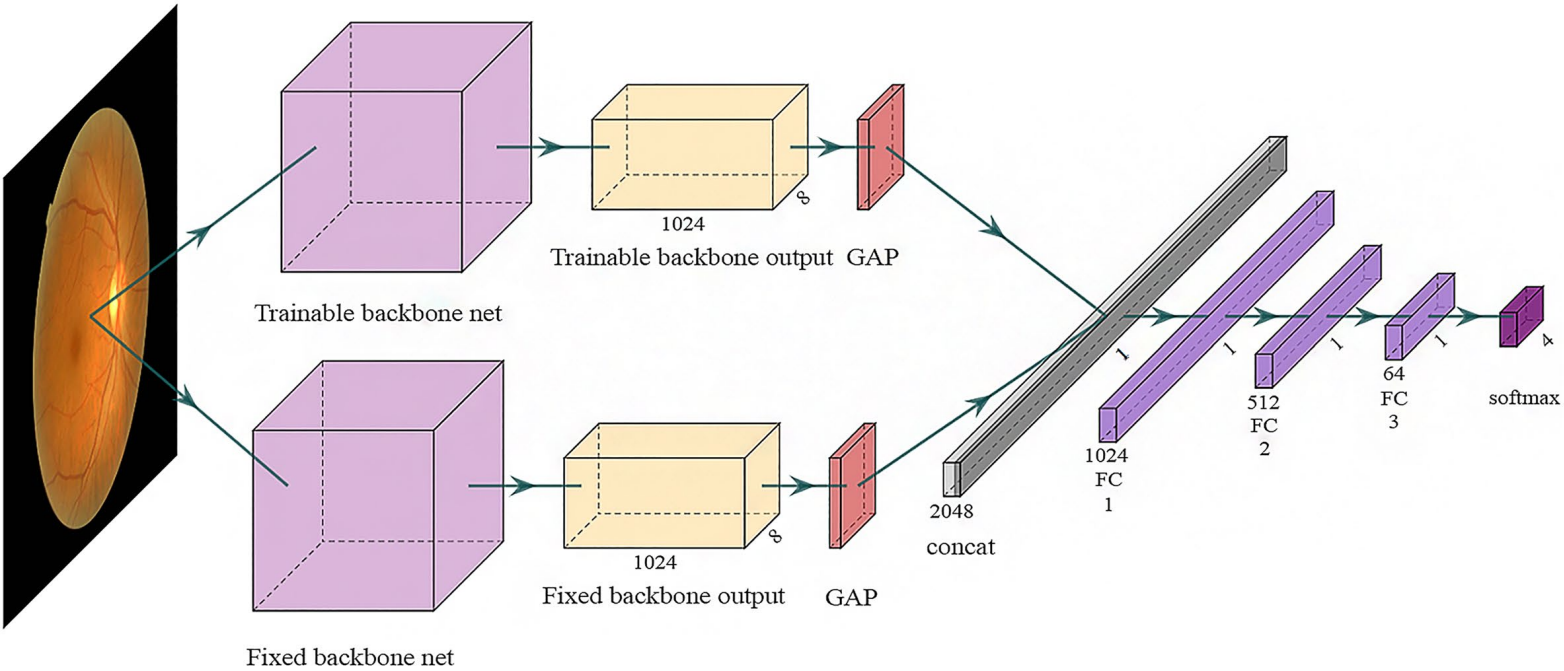
Model architecture for classification. The trainable backbone network (Upside) was updated during training to extract specific features for the optic disc. In contrast, the fixed backbone network (Downside) remained frozen during training, extracting general features for image classification.

We utilized well-established and high-performing deep-learning (DL) algorithms as pre-trained backbone networks, specifically VGG16 and VGG19^24^ as well as DenseNet121^25^. These algorithms have consistently shown excellent performance in medical computer vision tasks^23,26^. We initialized them with ImageNet-pre-trained weights. We adopted the two-pathway approach where a DL algorithm have two pathways of trainable and fixed encoders (Fig. 2), because it has been demonstrated to improve both the performance and convergence speed^27^. Especially in the classification of optic disc tilt, this two-pathway approach tends to enhance sensitivity performance without compromising specificity and accuracy^28^. The trainable backbone network and the fully connected (FC) block underwent updates during the training process. In contrast, the fixed backbone network remained unchanged throughout the training process. The FC block featured a linear layer, batch normalization, an activation function of rectified linear unit (ReLU), and dropout. The outputs of two backbone networks went respectively through global average pooling (GAP) before combined.

### Development and test of four-class classification model

A stratified random hold-out method was used to split the whole data at the subject level into two independent datasets with a ratio of 80% and 20%: (a) development dataset for training and internal validation, and (b) (external) test dataset for testing. The stratification of outcome classes was considered to reduce the sparsity problem arising from class imbalance. During the model development process, five iterations of fivefold stratified cross-validation were performed on the development dataset to optimize DL parameters with the use of categorical cross-entropy and AdamW for loss and optimizing and to conduct internal validation for best model selection. Futher details on model training can be found in Supplementary information 3. In the model testing stage, we evaluated the classification models using the independent test dataset.

### Statistical analysis

We employed the one-versus-rest strategy to assess the diagnostic performance of multi-class classification models. The overall performance was measured by accuracy, the fraction of correct classification. We also computed sensitivity, specificity precision, F1 score and the area under the receiver-operating-characteristic curve (AUC) using a weighted average to address class imbalance. We applied the n-out-of-n bootstrap approach with replacement, and obtain 100 resamples of the independent test dataset to estimate the performance metrics with their standard errors and to derive *P*-values for comparing model performances between the non-tilted and tilted optic disc images. Statistical significance was declared if two-sided *P*-values < 0.05.

## Results

A total of 2507 fundus photographs were acquired from 2236 eyes of 1809 subjects (mean age of 46 ± 20 years; 53% men; all Koreans; 33.4% with photographs of both eyes). Among all photographs (40.3% with tilted optic discs), 1,671 (66.7%) were annotated as ‘normal’ class whereas 477 (19.0%), 245 (9.8%), and 114 (4.5%) were diagnosed as classes of glaucomatous optic disc change, optic disc pallor, and optic disc swelling, respectively. Table 1 shows the distribution of classes in the non-tilted and tilted disc images.

**Table 1:** Distribution of outcome classes according to the tilt status in the development and test datasets.

Development dataset		All (n = 2005, k = 1555)	Non-tilted disc (n = 1198, k = 992)	Tilted disc (n = 807, k = 640)
Class, n (%)	Normal	1336 (66.6)	749 (62.5)	587 (72.7)
	Glaucoma	382 (19.1)	230 (19.2)	152 (18.8)
	Optic disc pallor	196 (9.8)	141 (11.8)	55 (6.8)
	Optic disc swelling	91 (4.5)	78 (6.5)	13 (1.6)

Test dataset		All (n = 502, k = 464)	Non-tilted disc (n = 299, k = 282)	Tilted disc (n = 203, k = 189)
Class, n (%)	Normal	335 (66.7)	179 (59.9)	156 (76.8)
	Glaucoma	95 (18.9)	63 (21.1)	32 (15.8)
	Optic disc pallor	49 (9.8)	38 (12.7)	11 (5.4)
	Optic disc swelling	23 (4.6)	19 (6.4)	4 (2.0)

This table presents the number (percent) of images annotated with each outcome class in each dataset category, illustrating the allocation of data for model training and evaluation. n = numbers of images. k = numbers of patients. Note that, some of the collected images belong to the same individual patients.

### Model performance

The metric results of the test data are presented in Table 2. For models trained and tested using the all dataset, the AUC values were 0.983 ± 0.002, 0.984 ± 0.006, and 0.982 ± 0.003 for VGG16, VGG19, and DenseNet121, respectively. When using the non-tilted disc dataset, the AUC values were 0.988 ± 0.002, 0.991 ± 0.003, and 0.986 ± 0.003. For the tilted disc dataset, the AUC values were 0.924 ± 0.046, 0.928 ± 0.017, and 0.935 ± 0.008.

**Table 2:** Performance metrics assessed on the test datasets for DL-based classification models with different backbone networks.

Development dataset	Test dataset	Backbone network	Accuracy	Precision	F1 score	AUC
All	All	VGG19	0.945 ± 0.004	0.946 ± 0.003	0.945 ± 0.003	0.983 ± 0.002
		VGG16	0.946 ± 0.007	0.947 ± 0.006	0.946 ± 0.006	0.984 ± 0.006
		Dense121	0.949 ± 0.004	0.951 ± 0.003	0.949 ± 0.004	0.982 ± 0.003
All	Non-tilted disc	VGG19	0.951 ± 0.003	0.952 ± 0.002	0.951 ± 0.003	0.989 ± 0.002
		VGG16	0.957 ± 0.008	0.958 ± 0.008	0.957 ± 0.008	0.991 ± 0.005
		Dense121	0.956 ± 0.006	0.959 ± 0.006	0.956 ± 0.006	0.985 ± 0.003
All	Tilted disc	VGG19	0.936 ± 0.006	0.940 ± 0.006	0.935 ± 0.006	0.969 ± 0.005
		VGG16	0.930 ± 0.010	0.933 ± 0.010	0.930 ± 0.010	0.969 ± 0.009
		Dense121	0.938 ± 0.004	0.942 ± 0.004	0.937 ± 0.005	0.977 ± 0.011
Non-tilted disc	Non-tilted disc	VGG19	0.945 ± 0.006	0.946 ± 0.006	0.945 ± 0.007	0.988 ± 0.002
		VGG16	0.944 ± 0.009	0.945 ± 0.009	0.943 ± 0.009	0.991 ± 0.003
		Dense121	0.944 ± 0.007	0.945 ± 0.007	0.944 ± 0.007	0.986 ± 0.003
Non-tilted disc	Tilted disc	VGG19	0.891 ± 0.028	0.903 ± 0.009	0.894 ± 0.020	0.927 ± 0.019
		VGG16	0.886 ± 0.019	0.902 ± 0.009	0.890 ± 0.011	0.922 ± 0.020
		Dense121	0.915 ± 0.007	0.926 ± 0.006	0.918 ± 0.006	0.944 ± 0.008
Tilted disc	Non-tilted disc	VGG19	0.878 ± 0.022	0.874 ± 0.040	0.869 ± 0.031	0.950 ± 0.013
		VGG16	0.886 ± 0.010	0.888 ± 0.012	0.878 ± 0.010	0.951 ± 0.015
		Dense121	0.891 ± 0.011	0.904 ± 0.009	0.884 ± 0.013	0.957 ± 0.008
Tilted disc	Tilted disc	VGG19	0.923 ± 0.009	0.922 ± 0.012	0.921 ± 0.009	0.924 ± 0.046
		VGG16	0.914 ± 0.009	0.913 ± 0.014	0.912 ± 0.011	0.928 ± 0.017
		Dense121	0.918 ± 0.007	0.917 ± 0.007	0.915 ± 0.008	0.935 ± 0.008

AUC = area under the curve.

The table displays the means ± standard errors of accuracy, precision, F1 score and the AUC values for classification models developed with VGG19, VGG16, and Dense121 using different pairs of development and test datasets.

The precision, F1 score, and AUC were computed using a weighted average to address class imbalance issues.

**Figure 3:**
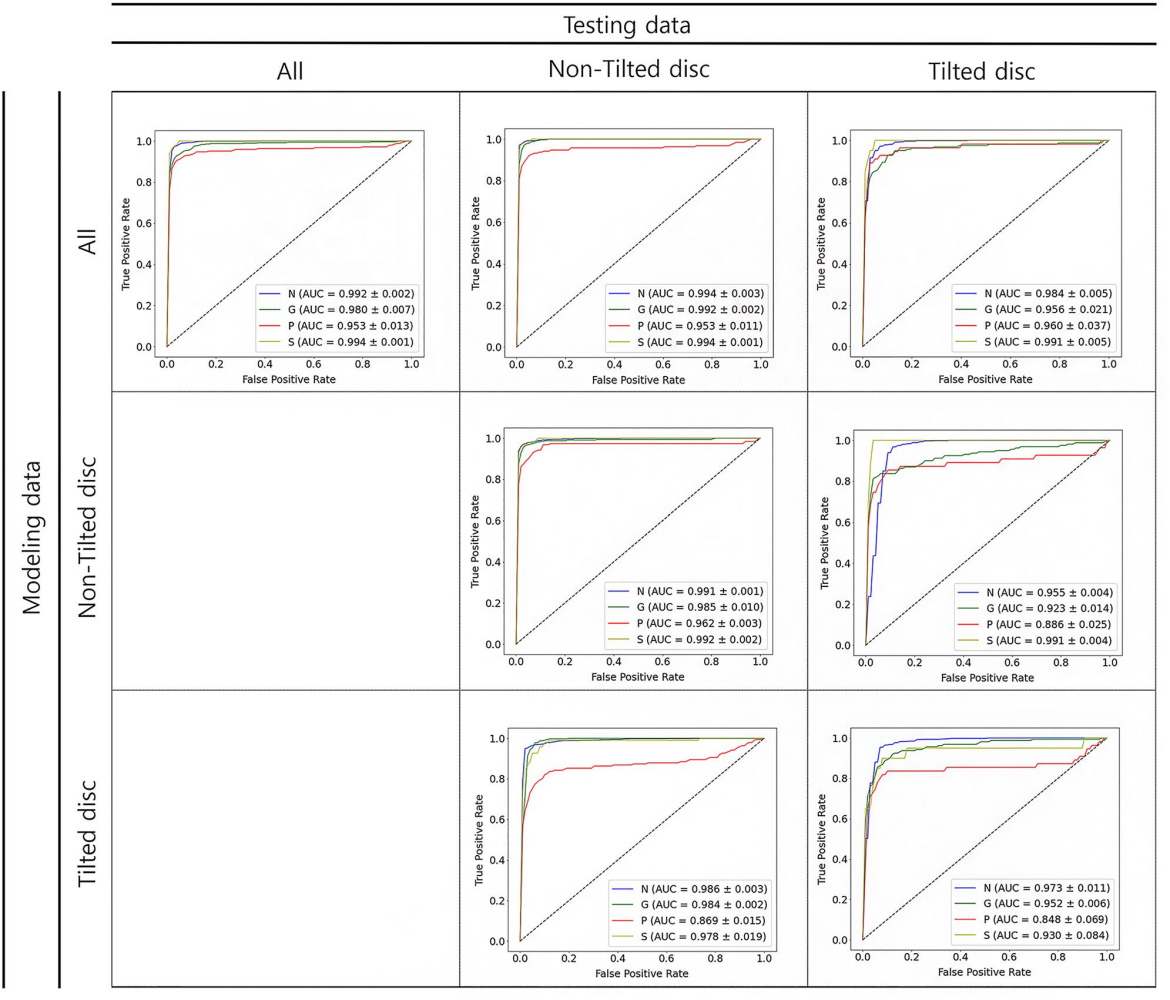
Receiver operating characteristics curves of DenseNet121 according to the dataset used. The columns represent the testing data categories: All, Non-Tilted disc, and Tilted disc. The rows indicate the modeling data used: All, Non-Tilted disc, and Tilted disc. Each graph showcases the True Positive Rate versus the False Positive Rate with the Area Under the Curve (AUC) values specified for each classification category. N = normal, G = glaucoma, P = optic disc pallor, S = optic disc swelling, Train: training data, Test: test data, AUC: area under the ROC curve.

Models trained with either the entire dataset or the non-tilted disc dataset exhibited better F1 scores when tested on the non-tilted disc dataset compared to the tilted disc dataset. Conversely, the model trained on the tilted disc dataset demonstrated a higher F1 score when tested on the tilted disc dataset than on the non-tilted disc dataset. In terms of AUC, regardless of the training dataset, models performed better when tested on the non-tilted disc dataset, as shown in Table 2 and Fig. 3.

**Figure 4:**
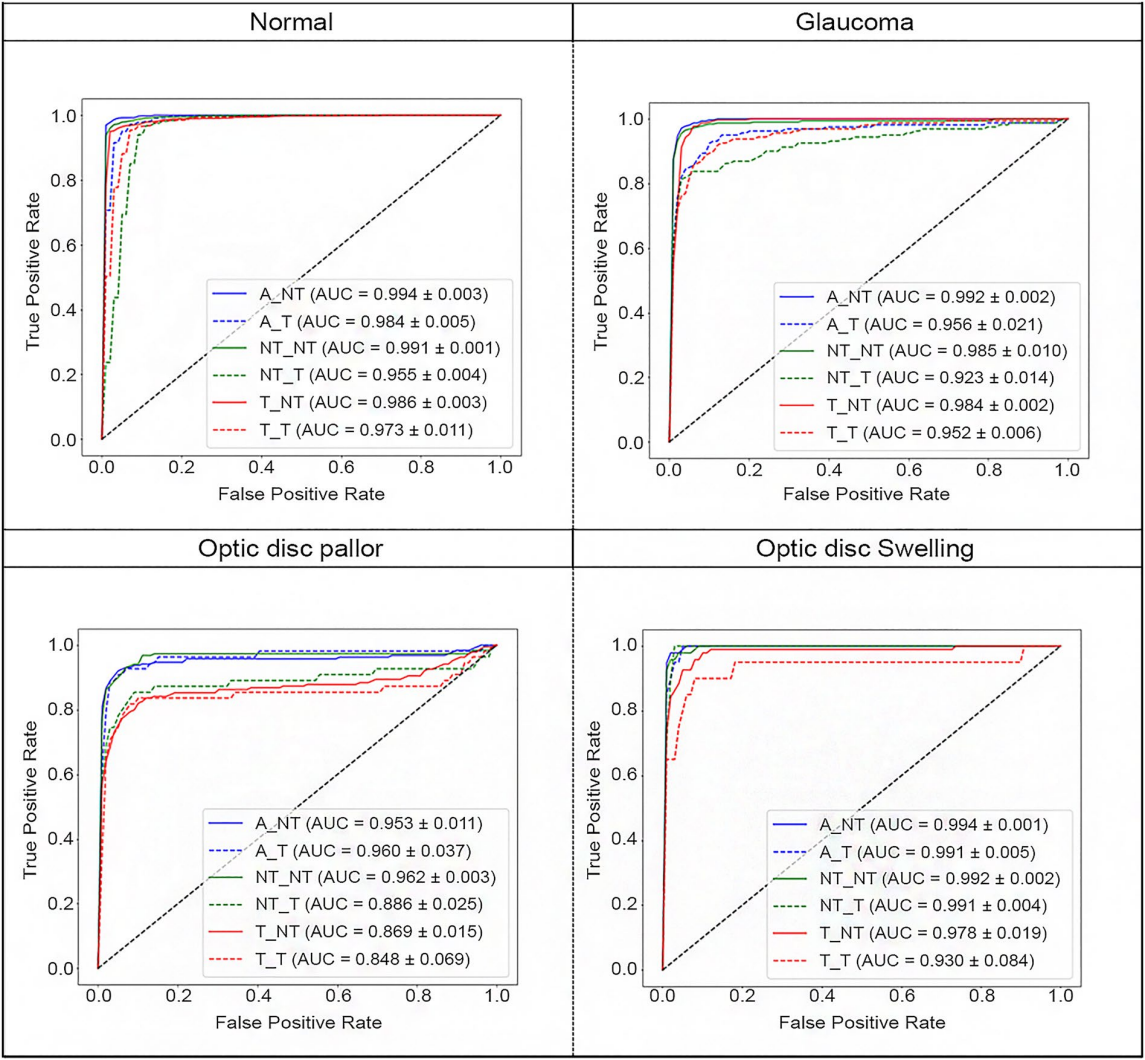
Receiver operating characteristics curves of DenseNet121 according to optic nerve disease. Receiver Operating Characteristic (ROC) curves for DenseNet121 stratified by optic nerve diseases: Normal, Glaucoma, Optic Disc Pallor, and Optic Disc Swelling. The curves represent various combinations of modeling and testing datasets. A = all, T = tilted disc, NT = non-tilted disc. A_NT: modeling using the all dataset and testing using the non-tilted disc dataset. A_T: modeling using the all dataset and testing using the tilted disc dataset. NT_NT: modeling using the non-tilted disc dataset and testing using the non-tilted disc dataset. NT_T: modeling using the non-tilted disc dataset and testing using the tilted disc dataset. T_NT: modeling using the tilted disc dataset and testing using the non-tilted disc dataset. T_T: modeling using the tilted disc dataset and testing using the tilted disc dataset. AUC: area under the ROC curve.

Figure 4 illustrates the ROC curve of DenseNet121 for different optic nerve diseases. For normal discs, modeling with the entire dataset and testing with the non-tilted disc (A_NT) yielded the highest AUC performance. Conversely, using the tilted disc dataset for both modeling and testing (T_T) resulted in the lowest AUC performance. In cases of glaucoma, A_NT and NT_T had the best and worst outcomes, respectively. With optic disc pallor, NT_NT and T_T showed the top and bottom performance, in that order. Similarly, for optic disc swelling, A_NT and T_T showed the best and worst results, respectively.

Table 3 presents the bootstrap test outcomes for each label. For normal discs, the non-tilted disc model significantly outperformed the tilted-disc model in sensitivity and F1 scores. There was no statistically significant difference in accuracy, specificity, or precision between non-tilted and tilted discs. In glaucoma, the non-tilted disc model showed significantly better performance than the tilted-disc model in accuracy, specificity, and precision. There were no statistically significant differences in sensitivity and F1 scores. In optic disc pallor, the non-tilted disc model showed significantly better performance than the tilted-disc model in specificity. There were no statistically significant differences in accuracy, sensitivity, precision, or F1 scores. In optic disc swelling, the non-tilted disc model showed significantly better performance than the tilted-disc model in specificity. There were no statistically significant differences in accuracy, sensitivity, precision, or F1 scores. The results of other backbone models are reported in Supplementary information 1. In the normal group, the non-tilted disc model showed better sensitivity than the tilted disc model. In contrast, in the pathologic groups of glaucoma, disc pallor, and disc swelling, the non-tilted disc model showed better specificity than the tilted disc model.

**Table 3:** Comparison in performance metrics of the DenseNet121-based classification model for each outcome class between the non-tilted vs. tilted disc images using the test dataset.

Outcome class	Metric	Non-tilted disc	Tilted disc	*P-v*alue
Normal	Accuracy	0.962 ± 0.008	0.945 ± 0.009	0.05
	Sensitivity	0.967 ± 0.015	0.841 ± 0.032	< 0.01
	Specificity	0.958 ± 0.013	0.976 ± 0.009	0.15
	Precision	0.940 ± 0.017	0.914 ± 0.028	0.23
	F1 score	0.953 ± 0.009	0.876 ± 0.020	< 0.01
Glaucoma	Accuracy	0.962 ± 0.009	0.939 ± 0.008	0.01
	Sensitivity	0.964 ± 0.013	0.965 ± 0.010	0.44
	Specificity	0.955 ± 0.037	0.798 ± 0.044	0.02
	Precision	0.988 ± 0.010	0.962 ± 0.008	0.03
	F1 score	0.975 ± 0.006	0.964 ± 0.005	0.06
Optic disc pallor	Accuracy	0.960 ± 0.008	0.960 ± 0.009	0.43
	Sensitivity	0.981 ± 0.012	0.980 ± 0.010	0.44
	Specificity	0.820 ± 0.058	0.608 ± 0.133	0.03
	Precision	0.974 ± 0.008	0.978 ± 0.007	0.37
	F1 score	0.977 ± 0.005	0.979 ± 0.005	0.40
Optic disc swelling	Accuracy	0.988 ± 0.005	0.985 ± 0.004	0.31
	Sensitivity	0.994 ± 0.005	0.999 ± 0.002	0.38
	Specificity	0.899 ± 0.055	0.275 ± 0.189	< 0.01
	Precision	0.993 ± 0.004	0.986 ± 0.004	0.10
	F1 score	0.994 ± 0.003	0.992 ± 0.002	0.31

The table displays the means ± standard errors of accuracy, sensitivity, specificity, precision and F1 score for the Dense121-based classification model in predicting each outcome class. *P*-value is a significance probability for testing the mean difference in each performance metric between classification models developed with the non-tilted vs. tilted disc images, and was derived from 100 bootstrap resamples of the test dataset.

## Discussion

Grossniklaus et al. reported that 37.7% of the pathologic findings in enucleated myopic eyes had a tilted appearance of the optic disc, with the retina falling short of the optic disc on one side and the retinal pigment epithelium and choroid extending over a portion of the optic disc on the other side^29^. It has been suggested to be an acquired feature in myopic eyes, with progressive tilting occurring between 7 and 9 years of age^30^. Because standard fundus photography and the normative database of ophthalmologic instruments rely on data from normal eyes with no or low myopia, imaging a distorted optic nerve head with moderate-to-high myopia that is structurally different from non-myopic populations may lead to critical misdiagnoses^31–33^. Hence, the ophthalmologist often makes subjective decisions by combining the results of various ophthalmologic tests in diagnosing those patients with myopia rather than using a consistent diagnostic rule. In addition, myopia itself was reported to be associated with numerous serious ophthalmic diseases such as macular degeneration, retinal detachments, and optic neuropathies, including glaucoma and other forms of optic neuropathy^32,34–37^. Several previous studies on deep learning algorithms detecting optic nerve head changes reported that the coexistence of a high degree of myopia caused misclassifications^18,38^. However, The extent and manner of their impact on the system's accuracy have not been assessed.

In this study, we developed deep learning-based optic disc appearance classification systems using fundus photographs with and without tilted optic discs^39^, and observed that a tilted disc adversely affected the performance of the optic disc classification systems. DenseNet121 trained and tested using the all-subject data showed accuracy, precision, F1 scores, and AUC of 0.949 ± 0.004, 0.951 ± 0.003, 0.949 ± 0.004, and 0.982 ± 0.003, respectively. DenseNet121 trained and tested using the non-tilted disc data showed 0.945 ± 0.007, 0.945 ± 0.007, 0.945 ± 0.007, and 0.986 ± 0.003 for accuracy, precision, F1 scores, and AUC, while that using the tilted disc data showed 0.918 ± 0.007, 0.917 ± 0.007, 0.915 ± 0.008, and 0.935 ± 0.008, respectively. In all three tested models trained with the same dataset, the accuracy, precision, F1 scores, and AUC for tilted discs images were lower by 0.026, 0.028, 0.028, and 0.059, respectively than those for non-tilted disc images.

In the normal group, accuracy, sensitivity, precision, and F1 score were marginally lower by 0.015, 0.095, 0.040, and 0.069 respectively for tilted-disc images compared to non-tilted ones. However, specificity for tilted-disc images exhibited a slight increase of 0.009 compared to non-tilted images. Regarding glaucomatous optic disc changes, all measured metrics showed reduced performance for tilted discs when compared to non-tilted ones. Similarly, for optic disc pallor, all metrics except precision were lower for tilted discs, with the F1 score showing no significant difference. Regarding optic disc swelling, all metrics except sensitivity were lower for tilted discs, where sensitivity showed a slight improvement.

These observations represent the average metric outcomes across the VGG16, VGG19, and DenseNet121 models. Notably, the presence of tilted discs generally hindered the performance of optic disc classification systems in our experiments. These findings align with prior clinical studies indicating that atypical disc appearances, such as tilted discs, could complicate differential diagnoses, resembling glaucomatous changes^40–44^

Instances have been reported where a tilted disc with an increased cup-to-disc ratio was misdiagnosed as normal-tension glaucoma^45^. An excavated disc, commonly observed in glaucoma, is also found in tilted discs, ischemic neuropathy, and compressive neuropathy, demanding comprehensive differential diagnostics before concluding an optic nerve disease diagnosis^42^.

Moreover, tilted optic discs, with an elevated nasal disc while being posteriorly displaced or blurred, often mimic optic disc swelling. Conversely, a tilted disc accompanied by mild papilledema might be mistaken as pseudopapilledema^43^. Studies reveal that interpreting fundus photographs can result in errors, especially in identifying mild optic nerve swelling from pseudo-disc edema or a normal nerve^16^. Recently, the emergence of peripapillary hyperreflective ovoid mass-like structures (PHOMS) on optical coherence tomography has accentuated the importance of accurate optic nerve classification^46,47^. PHOMS, assumed to be herniating nerve fibers above the Bruch membrane layer linked with myopic shift, is suggested to be mediated by optic disc tilt in its development^48^. Given that PHOMS presents as an elevated and blurred disc during fundoscopic examination, it is commonly misdiagnosed as optic disc swelling^47–49^. Classifying the optic nerve becomes more complex when PHOMS accompanies a significantly tilted optic disc and pathologic optic nerve changes.

Previous studies conducted by Li et al.^18^, Yang et al.^12^, Liu et al.^38^ and Hemelings et al.^50^ pointed out the coexistence of myopic optic disc changes as a major cause of false results in detecting glaucomatous optic nerve changes when using a deep learning system. Li et al.^18^ first reported that the coexistence of high or pathologic myopia was the most common cause of false-negative results for detecting glaucomatous optic nerve changes using a deep learning system. In the same context, Yang et al. reported that the most common reasons for false-positive cases of glaucomatous optic neuropathy were extensive areas of peripapillary atrophy and tilted optic discs in deep learning algorithms^12^. Liu et al.^38^ also reported that the main reason for both false-negative and false-positive diagnoses by GD-CNN and manual grading was high or pathologic myopia, which was characterized by peripapillary atrophy and shallow cups, and tilting, torsion, or both of the optic disc. Hemelings suggested that a major cause of false positives and negatives in glaucoma detection using deep learning was myopia with an induced skew of the optic cup^50^. Our study confirmed that tilted optic disc appearance significantly reduced diagnostic accuracy, sensitivity, or specificity for optic disc classification using an artificial intelligence deep learning system, not only for glaucomatous changes but also for other types of optic nerve head changes such as optic disc pallor and swelling, and even confirming normal optic disc morphology. To create the ground set of an ophthalmologic diagnostic system, the precedence of tilted disc classification may be important. The significant alteration in the performance in the group with tilted optic disc changes should be contemplated when developing automatic algorithms for optic disc classification.

This study had several limitations. First, a tilted disc was defined using only fundus photographs. The integration of variables from other device outputs, including optical coherence tomography, might be ideal for the definition of a tilted optic disc^4,40,51^.

Second, the proportion of each class was not equal in our data set. Especially, there were scarcity of cases featuring tilted optic discs within the optic disc pallor and swelling categories. Indeed, the incidence of swelling and pallor of the optic disc are very low compared to that of the glaucomatous optic disc, making it difficult to conduct studies for evaluating images from patients with optic disc pallor and swelling. Nevertheless, the differential diagnosis of optic disc swelling and pallor with glaucoma is extremely important because of different treatment approaches. Detecting these conditions can pose challenges^52,53^ especially when assessments are carried out by non-ophthalmic medical professionals like neurologists and general practitioners^54^. Importantly, recognizing them often requires prompt and comprehensive investigations, including brain imaging and cerebrospinal fluid analysis, to identify potential life-threatening or vision-threatening conditions. In addition, any delay in identifying such conditions could lead to permanent visual impairment and neurological complications. That was the reason why we conducted thorough analysis of disc pallor and swelling in tilted optic discs, despite their rarity in cases. We aimed for our deep learning-based optic disc appearance system to enhance the accuracy of diagnosing optic disc diseases, regardless of their prevalence.

Third, the potential variance in classification difficulty among different cases may affect the performance of our deep learning-based optic disc classification system because we did not classify the severity of optic disc swelling and pallor for our analysis. Nonetheless, our study predominantly investigated the impact of optic disc tilt on deep learning-based optic disc classification. The primary objective of this study was to improve the differentiation between normal optic discs and those exhibiting signs of swelling or pallor, with a particular focus on optic disc with tilt and especially in cases of mild optic disc swelling or pallor. To ensure a comprehensive analysis, we incorporated a wide spectrum of cases varying in the severity of optic disc swelling and pallor from our institution, spanning from 2007 to 2021. We believe our methodology in fundus photograph collection and analysis aligns with the purpose of this study.

Fourth, potential errors might have arisen in our deep learning-based optic disc appearance classification system due to the heterogeneity of our dataset, particularly regarding centration, angle of view, and image pixel sizes. To mitigate these errors, we eliminated unnecessary margins around the optic disc and obscured extraneous information in the images, such as patient ID, date, and shooting angles. Images of various resolution and angle of view underwent this manual adjustment, as depicted in Fig. 1. However, the diverse nature of our dataset, encompassing various fundus photography settings, may facilitate the creation of an optic disc appearance classification system that is widely applicable across different types of fundus photographs encountered in clinical practice.

Fifth, there was no external validation in this study. To properly assess clinical performance in our deep learning systems, external validation^55^ with larger datasets must be performed to adequately represent the manifestations of a broad spectrum of optic neuropathies in various clinical settings^56,57^. Finally, our deep learning model should be validated in different ethnicities because only the Korean population was recruited in this study.

In conclusion, we developed deep learning-based optic disc appearance classification systems using the fundus photographs of patients with and without tilted optic discs. The classification performance was lower in tilted discs than in non-tilted discs, suggesting the need to identify and adjust for the effect of optic disc tilt in future development of the optic disc classification algorithm.

### Supplementary Information

Supplementary Information 1.

Supplementary Information 2.

Supplementary Information 3.

## Data Availability

The datasets generated and/or analyzed during the current study are not publicly available due to Samsung Medical Center policy. But are available from the corresponding author on reasonable request.
