# Supplementary material for: Deep learning-based optic disc classification is affected by optic-disc tilt
Source: Sci Rep. 2024 Jan 4;14:498. doi: 10.1038/s41598-023-50256-4 (PMC10767025; doi:10.1038/s41598-023-50256-4)
Supplement: Supplementary file 1 — Supplementary Information 1. [file 41598_2023_50256_MOESM1_ESM.pdf]

Supplementary information 1. Comparison in performance metrics of the VGG19- and VGG16-based classification models for each outcome class between the non-tilted vs. tilted disc images using the test dataset.

Backbone network	Outcome class	Metric	Non-tilted disc	Tilted disc	*P*-value
VGG19	Normal	Accuracy	0.964±0.008	0.951±0.010	0.14
		Sensitivity	0.962±0.023	0.885±0.042	0.04
		Specificity	0.966±0.012	0.971±0.010	0.31
		Precision	0.950±0.016	0.904±0.028	0.06
		F1 score	0.956±0.011	0.894±0.023	0.01
	Glaucoma	Accuracy	0.965±0.008	0.940±0.009	0.02
		Sensitivity	0.968±0.012	0.966±0.012	0.42
		Specificity	0.952±0.028	0.800±0.067	0.01
		Precision	0.987±0.007	0.963±0.012	0.05
		F1 score	0.977±0.005	0.964±0.005	0.03
	Optic disc pallor	Accuracy	0.964±0.007	0.958±0.010	0.32
		Sensitivity	0.985±0.009	0.976±0.012	0.26
		Specificity	0.822±0.066	0.650±0.149	0.18
		Precision	0.974±0.009	0.980±0.008	0.31
		F1 score	0.980±0.004	0.978±0.006	0.4
	Optic disc swelling	Accuracy	0.983±0.006	0.980±0.007	0.39
		Sensitivity	0.991±0.006	0.993±0.007	0.45
		Specificity	0.869±0.080	0.352±0.191	0
		Precision	0.991±0.005	0.987±0.004	0.28
		F1 score	0.991±0.003	0.990±0.003	0.39
VGG16	Normal	Accuracy	0.967±0.008	0.951±0.009	0.08
		Sensitivity	0.965±0.021	0.883±0.041	0.01
		Specificity	0.968±0.010	0.971±0.011	0.42
		Precision	0.953±0.013	0.905±0.032	0.07
		F1 score	0.959±0.010	0.892±0.021	0
	Glaucoma	Accuracy	0.967±0.008	0.940±0.009	0.02
		Sensitivity	0.973±0.011	0.965±0.012	0.33
		Specificity	0.945±0.029	0.803±0.075	0.03
		Precision	0.985±0.008	0.964±0.013	0.07
		F1 score	0.979±0.005	0.964±0.005	0.02
	Optic disc pallor	Accuracy	0.963±0.007	0.959±0.012	0.41
		Sensitivity	0.983±0.008	0.976±0.015	0.36

	Specificity	0.829±0.068	0.667±0.140	0.19
	Precision	0.975±0.009	0.981±0.008	0.34
	F1 score	0.979±0.004	0.978±0.006	0.47
Optic disc swelling	Accuracy	0.985±0.005	0.983±0.006	0.43
	Sensitivity	0.991±0.005	0.995±0.006	0.26
	Specificity	0.895±0.068	0.365±0.228	0
	Precision	0.993±0.005	0.987±0.004	0.18
	F1s score	0.992±0.003	0.991±0.003	0.43

The table displays the means ± standard errors of accuracy, sensitivity, specificity, precision and F1 score for the VGG-based classification models in predicting each outcome class. *P*-value is a significance probability for testing the mean difference in each performance metric between classification models developed with the non-tilted vs. tilted disc images, and was derived from 100 bootstrap resamples of the test dataset.
