# Supplementary material for: Deep learning-based optic disc classification is affected by optic-disc tilt
Source: Sci Rep. 2024 Jan 4;14:498. doi: 10.1038/s41598-023-50256-4 (PMC10767025; doi:10.1038/s41598-023-50256-4)
Supplement: Supplementary file 2 — Supplementary Information 2. [file 41598_2023_50256_MOESM2_ESM.pdf]

Supplementary information 2. Confusion matrices for four-class classification models with different backbone networks (N: normal class, G: glaucomatous optic disc change, P: optic disc pallor, S: optic disc swelling).

Model	True label	All: Predicted N	All: Predicted G	All: Predicted P	All: Predicted S	Non-Tilted disc: Predicted N	Non-Tilted disc: Predicted G	Non-Tilted disc: Predicted P	Non-Tilted disc: Predicted S	Tilted disc: Predicted N	Tilted disc: Predicted G	Tilted disc: Predicted P	Tilted disc: Predicted S
DenseNet121	N	330	3	2	0	173	3	2	1	152	4	0	0
	G	4	88	3	0	0	61	2	0	4	26	2	0
	P	2	4	43	0	2	2	33	1	1	3	7	0
	S	3	0	0	20	1	0	0	18	1	1	0	2
VGG16	N	332	2	1	0	175	2	1	1	152	3	1	0
	G	4	84	7	0	0	61	2	0	3	27	2	0
	P	2	3	43	1	2	4	31	1	0	4	7	0
	S	1	0	0	22	2	0	0	17	1	1	0	2
VGG19	N	332	2	1	0	175	1	1	2	152	3	1	0
	G	4	87	4	0	0	61	2	0	3	27	2	0
	P	1	4	43	1	2	3	32	1	0	3	8	0
	S	3	0	1	19	2	0	0	17	1	1	0	2
