# Supplementary material for: Deep learning-based optic disc classification is affected by optic-disc tilt
Source: Sci Rep. 2024 Jan 4;14:498. doi: 10.1038/s41598-023-50256-4 (PMC10767025; doi:10.1038/s41598-023-50256-4)
Supplement: Supplementary file 3 — Supplementary Information 3. [file 41598_2023_50256_MOESM3_ESM.pdf]

Supplementary information 3. Technical details in the model training

The training was performed for 100 epochs for each experiment, and a batch size of 64 was used. We used categorical cross-entropy and AdamW (17) for loss and optimizing, respectively. The hyperparameters used for optimizing were 0.9, 0.999, and 1e-6 for beta_1, beta_2, and epsilon respectively. The first 10% of the entire training step was used as the warm-up stage. After the warm-up stage, the learning rate was set as 1e-4 and decayed linearly to zero.
